# Gradual Disturbances of the Amplitude of Low-Frequency Fluctuations (ALFF) and Fractional ALFF in Alzheimer Spectrum

**DOI:** 10.3389/fnins.2018.00975

**Published:** 2018-12-20

**Authors:** Liu Yang, Yan Yan, Yonghao Wang, Xiaochen Hu, Jie Lu, Piu Chan, Tianyi Yan, Ying Han

**Affiliations:** ^1^Department of Neurology, Xuanwu Hospital, Capital Medical University, Beijing, China; ^2^School of Life Science, Beijing Institute of Technology, Beijing, China; ^3^Department of Psychiatry and Psychotherapy, Medical Faculty, University of Cologne, Cologne, Germany; ^4^Department of Radiology, Xuanwu Hospital, Capital Medical University, Beijing, China; ^5^Beijing Institute of Geriatrics, Beijing, China; ^6^National Clinical Research Center for Geriatric Disorders, Beijing, China; ^7^Center of Alzheimer’s Disease, Beijing Institute for Brain Disorders, Beijing, China

**Keywords:** Alzheimer’s disease, subjective cognitive decline, amnestic mild cognitive impairment, dementia, resting-state functional MRI, ALFF, fALFF, classifier

## Abstract

**Background:** Alzheimer’s disease (AD) is a common neurodegenerative disease in which the brain undergoes alterations for decades before symptoms become obvious. Subjective cognitive decline (SCD) have self-complain of persistent decline in cognitive function especially in memory but perform normally on standard neuropsychological tests. SCD with the presence of AD pathology is the transitional stage 2 of Alzheimer’s continuum, earlier than the prodromal stage, mild cognitive impairment (MCI), which seems to be the best target to research AD. In this study, we aimed to detect the transformational patterns of the intrinsic brain activity as the disease burden got heavy.

**Method:** In this study, we enrolled 44 SCD, 55 amnestic MCI (aMCI), 47 AD dementia (d-AD) patients and 57 normal controls (NC) in total. A machine learning classification was utilized to detect identification accuracies between groups by using ALFF, fALFF, and fusing ALFF with fALFF features. Then, we measured the amplitude of the low-frequency fluctuation (ALFF) and fractional ALFF (fALFF) levels in three frequency bands (classic: 0.01–0.1 Hz; slow-5: 0.01–0.027 Hz; and slow-4: 0.027–0.073 Hz) and compared alterations in patients with NC.

**Results:** In the machine learning verification, the identification accuracy of SCD, aMCI, d-AD from NC was higher when fused ALFF and fALFF features (76.44, 81.94, and 91.83%, respectively) than only using ALFF or fALFF features. Several brain regions showed significant differences in ALFF/fALFF within these bands among four groups: brain regions presented decreasing trend of values, including the Cingulum_Mid_R (aal), bilateral inferior cerebellum lobe, bilateral precuneus, and the Cingulum_Ant_R (aal); increasing trend of values were detected in the Hippocampus_L (aal), Frontal_Mid_Orb_R (aal), Frontal_Sup_R (aal) and Paracentral_Lobule_R (aal) as disease progressed. The normalized ALFF/fALFF values of these features were significantly correlated with the neuropsychological test scores.

**Conclusion:** This study revealed gradual disturbances in intrinsic brain activity as the disease progressed: the normal objective performance in SCD may be dependent on compensation; as disease advanced, the cognitive function gradually impaired and decompensated in aMCI, severer in d-AD. Our results indicated that the ALFF and fALFF may help detect the underlying pathological mechanism in AD continuum.

**Clinical Trial Registration:** ClinicalTrials.gov, identifier NCT02353884 and NCT02225964.

## Introduction

Alzheimer’s disease (AD) is the most common progressive neurodegenerative disease of the elderly without a definite pathogenesis or effective treatment being found to decelerate the progression of this disorder, leading to poor outcomes and severe burdens to both the family and society (Scheltens et al., 2016). Explanations for the failure of drug clinical trials may be summarized by interventions during a stage of disease that is too late (McDade and Bateman, 2017). Thus, early detection of AD seems to be advantageous for the application of preventive means and may therefore help delay the progression. Clinical studies suggest the occurrence of very subtle cognitive alterations that are detectable years before meeting the criteria for MCI, predicting the progression to d-AD (Sperling et al., 2011). SCD refers to those elderly people who self-report a persistent decline in cognition but perform normally on the standard assessment with a higher conversion risk to MCI or d-AD, which is the transitional stage 2 of Alzheimer’s continuum in the presence of AD pathology and seems to be the best target to research the underlying mechanism of AD (Sperling et al., 2011; Jessen et al., 2014; Mitchell et al., 2014; Jack et al., 2018; Jessen and Rodriguez Nee Then, 2018).

Neuroimaging techniques could help detect structural and functional brain abnormalities at an early stage before objective deficits are detectable. The rs-fMRI is a promising non-invasive functional imaging technique to measure spontaneous brain activities *in vivo* and helps detect the intrinsic brain functional architecture under normal and pathological conditions such as AD without performance confounders (Zhang and Raichle, 2010). It has been widely used to explore the neurophysiological mechanism and neural process of human cognition and to identify the functional integrity of brain networks supporting memory and other cognitive domains in AD (Sperling, 2011; Binnewijzend et al., 2012). The ALFF was introduced as a measure for the magnitude of LFOs of rs-fMRI. It is defined as the total power within the frequency range between 0.01 and 0.1 Hz and considered as an effective approach to detect the regional intensity of spontaneous fluctuations and to reflect spontaneous brain activity of the brain in the BOLD signal of the rs-fMRI (Fox and Raichle, 2007; Tomasi et al., 2013). Studies have indicated that this index may be used as a marker for disease states of the brain (Han et al., 2011; Chen et al., 2015; Wang et al., 2016). However, it can be impaired and influenced by non-neural physiological fluctuations such as respiration, cardiac action, and motion. The fALFF is the ALFF of a given frequency band as a fraction of the sum amplitudes across the whole frequency range. It is a normalized and modified index of ALFF that can improve the sensitivity and specificity for the detection of spontaneous brain activities by surpassing the physiological noise, especially in perivascular, periventricular and periaqueductal regions (Zou et al., 2008). However, it is less reliable than ALFF as a proportional measure (Zuo et al., 2010). These two indexes reflect different aspects of the LFOs amplitude: ALFF represents the strength of intensity of LFOs, while fALFF indexes the relative contribution of a specific LFOs to the entire frequency range (Zuo et al., 2010). They are both useful to characterize the physiology of AD, reveal intrinsic network disruption and are sensitive indexes to detect AD-related neurodegeneration (Han et al., 2011, 2012).

Rs-fMRI signals in the cortical and cistern areas may have different characteristics in the field of their power distribution in different frequency ranges (Zou et al., 2008). The independent frequency bands are generated by distinct oscillators with particular properties and physiological functions, and the pattern of intrinsic brain activity is sensitive to particular frequency bands (Buzsaki and Draguhn, 2004). Several studies have demonstrated that the pattern of disrupted LFOs in aMCI and d-AD is frequency-dependent (Han et al., 2011; Wang et al., 2011; Liu et al., 2014). ALFF/fALFF in the slow-5 band seem to be more sensitive to changes in the DMN than the slow-4 band in aMCI (Han et al., 2011). There seems to be different patterns of disruption in the slow-5 band compared with the slow-4 band (Liu et al., 2014). Therefore, it is necessary to differentiate the frequency bands to further examine the specific alterations in distinct brain regions. In this study, we divided the frequency bands into three signals (classic frequency band: 0.01–0.1 Hz; slow-5: 0.01–0.027 Hz; and slow-4: 0.027–0.073 Hz) to detect diverse and comprehensive oscillation properties of the brain (Buzsaki and Draguhn, 2004; Zuo et al., 2010).

A gradual neurodegenerative processing seems to occur in AD. From the perspective of structural transformation, the WM degradation and GM atrophy in SCD was similar to aMCI and d-AD with slight extents (Meiberth et al., 2015; Cantero et al., 2016). With respect to functional alterations, previous studies have detected several brain regions with higher ALFF in SCD related to those exhibiting functional disruptions in MCI and d-AD, which may indicate a possible compensation mechanism in the early stage of AD (Sperling et al., 2009; Sun et al., 2016). Studies in aMCI and d-AD have identified both regions with decreased and increased ALFF/fALFF compared with the NC (Wang et al., 2011; Liu et al., 2014; Cai et al., 2017; Lin et al., 2017), suggesting an impairment and compensation concurrently exist in aMCI and d-AD (Qi et al., 2010; Liu et al., 2014). Thus, we wondered whether a special pattern of functional alterations is present throughout the course of AD.

Amplitude of low-frequency fluctuations and fALFF both have strengths and weaknesses, and they cannot substitute for each other in the detection of intrinsic brain activity. In the current study, we extracted mALFF and normalized fALFF (mfALFF) values of rs-fMRI in three frequency bands, and further utilized machine learning algorithms to construct a classifier to detect the clinical classification efficacy of ALFF, fALFF features, and the combination of them; explored regional differences in intrinsic activities among NC, SCD, aMCI, and d-AD groups; then detected brain regions with alterations, attempting to generalize the alterations of intrinsic brain activity patterns in the resting-state of the AD continuum and explain their behavioral deficiency. We hypothesized that (1) the classifier constructed by ALFF and fALFF features would get a high identification accuracy; (2) the values of mALFF/mfALFF may get changed in patient groups; (3) as the disease progressed, the alterations in mALFF/mfALFF values turned to be obvious and closely correlated with their cognitive levels.

## Materials and Methods

### Participants

A total of 220 right-hand, Han Chinese subjects were enrolled in this study from September 2009 to December 2015. Among them, 61 NC were recruited from the local community by advertisements. One hundred fifty-nine subjects with memory complaints were enlisted from the memory clinic of the Neurology Department of Xuanwu Hospital in Beijing, China, including 46 SCD, 60 aMCI, and 53 d-AD patients. The research was authorized by the Medical Research Ethics Committee and Institutional Review Board of Xuanwu Hospital (ClinicalTrials.gov identifier: NCT02353884 and NCT02225964). Each participant was provided with a written informed consent and signed it prior to any procedures. All subjects underwent a set of standardized clinical evaluations, including a medical history enquiry, neurological examination, and a suite of neuropsychological tests, which included the Chinese version of the MMSE, the Beijing version of MoCA (Lu et al., 2011), the AVLT (Guo et al., 2007), CDR (Morris, 1993), ADL, HIS, HAMD (Hamilton, 1960), and Center for Epidemiologic Studies depression scale (Dozeman et al., 2011). The diagnosis was made by experienced neurologists according to established guidelines. The NC must meet the following conditions: (a) no memory concerns; (b) MMSE (>19 for illiteracy, >22 for 1–6 educational years, >26 for more than 6 educational years) (Zhang et al., 1999) and MoCA scores (>13 for illiteracy, >19 for 1–6 educational years, >24 for more than 6 educational years) (Lu et al., 2011); and (c) CDR score of 0. SCD fulfilled the SCD research criteria proposed by the Subjective Cognitive Decline Initiative (SCD-I) (Jessen et al., 2014): (a) self-report persistent memory decline within the last 5 years compared with the previous normal status and confirmed by an informant; (b) normal range scores of MMSE and MoCA; and (c) CDR score of 0. The aMCI subjects were included based on the following items: (a) with or without self-perceived memory complaint and with informant complaints; (b) objectively impaired memory confirmed by MMSE (≤19 for illiteracy, ≤22 for 1–6 educational years, ≤26 for more than 6 educational years) and MoCA scores (≤13 for illiteracy, ≤19 for 1–6 educational years, ≤24 for more than 6 educational years); (c) clear-cut history of worsening cognition; (d) failure to meet the criteria for dementia according to the Diagnostic and Statistical Manual of Mental Disorders, Fourth Edition, revised (DSM-IV-R); and (e) CDR score of 0.5. The d-AD patients were diagnosed according to the National Institute of Aging-Alzheimer’s (NIA-AA) criteria for clinically probable AD (McKhann et al., 2011): (a) meeting criteria for dementia; (b) insidious and gradual onset (not sudden) over more than 6 months; (c) clear-cut history of worsening cognition; (d) initial and most prominent cognitive deficits evident in performance of the amnestic presentation or non-amnestic presentations; (e) hippocampal atrophy confirmed by structural MRI; and (f) CDR score ≥1.

The exclusion criteria for all subjects included: (a) a history of stroke (HIS score >4); (b) severe depression (HAMD score >24 or center for Epidemiological Studies Depression Scale score >21); (c) other central nervous system diseases that may cause cognitive decline (e.g., epilepsy, brain tumors, Parkinson’s disease, or encephalitis); (d) systemic diseases that could induce cognitive impairment (e.g., anthracemia, syphilis, thyroid dysfunction, or severe anemia, or HIV); (e) a history of psychosis or congenital mental growth retardation; (f) sever hypoplasia or dysacusis; (g) cognitive deficit caused by traumatic brain injury; (h) severe end-stage diseases or severe diseases in acute stages; or (i) those who could not complete neuropsychological tests or were contraindicated for MRI.

### Image Data Acquisition

All participants were imaged with a 3.0 Tesla MR imager (Siemens Magnetom Trio Tim MRI system, Germany) using a standard head coil. Cushions and earplugs were used to reduce subject motion and scanner noise. Before imaging, subjects were asked to keep their eyes closed and relaxed, but not to fall asleep and to move as little as possible during the imaging. The echo plane imaging sequence was applied to collect functional images. The scanning parameters were as follows: repetition time (TR) = 2000 ms, echo time (TE) = 40 ms, flip angle (FA) = 90°, field of view (FOV) = 240 mm × 240 mm, number of layers = 28, layer thickness = 4 mm, matrix = 64 × 64, voxel size = 3.75 mm × 3.75 mm × 4 mm, layer interval = 1 mm, bandwidth = 2232 Hz per pixel. The sequence lasted for 478 s, so each scan of a subject included 239 phases. In addition, a T1-weighted image was acquired as an anatomical reference. T1-weighted MR images were obtained by a 3D magnetization-prepared rapid gradient echo (MPRAGE) with the following parameters: slices = 176, TR = 1900 ms, TE = 2 ms, inversion time (TI) = 900 ms, FA = 9°, FOV = 224 mm × 256 mm, acquisition matrix = 448 × 512, no gap, and thickness = 1.0 mm.

### Image Data Preprocessing

Based on the MATLAB software platform, all images were processed using the static MR data processing toolkit *GRETNA v2.0.0*^1^. The image pre-processing steps consisted of the following. (1) The data for the first ten volumes were deleted to reduce the effect of magnetic field in homogeneity during the initial scan and to adapt subjects to the scanning environment. (2) Time correction was used to correct the difference in acquisition time between layers of a volume. (3) Head correction was performed by removing subjects who had large head movements. Each subject generated two types of figures: the translation diagram shows the translation of the head in the three directions, *X, Y*, and *Z*, and the rotation diagram shows the rotation angle of the three axes around *X, Y*, and *Z* in the experiment. Six-parameter motion regression estimates were used to calculate the framewise displacement (FD). In this study, subjects were deleted with maximum movements in translation > 3 mm or a rotation angle > 3°. (4) Space standardization: differences exist among human brains, both in shapes and volumes. To obtain the uniform coordinate system to describe the same anatomical location, we used spatial standardization so that the brains of different subjects were registered with the same standard space, Montreal Institute of Neurology, Standard Head Anatomical Template (MNI) space. In this study, structural images (T1 images) were used to register functional images to achieve spatial standardization of the subjects. A matrix was generated after registration and segmentation. The data of the matrix was applied to the functional images, which was used to realize the registration from the functional space of subjects to the standard space (MNI space). Finally, the functional images were then registered with the segmented structure images, and the resulting data were re-sampled to obtain functional data of 3 mm × 3 mm × 3 mm voxels. (5) Smoothing with a 4-mm full width at half maximum Gaussian kernel: smoothing can reduce the incomplete effects of registration so that the residuals are more consistent with the Gaussian distribution and improve the image signal-to-noise ratio. (6) Remove linear drift. (7) Regress out covariates including the global signal, WM signal, cerebrospinal fluid signal and Friston-24 parameters.

For normalization, the ALFF of each voxel is divided by the average ALFF of all voxels in the whole brain to obtain the mALFF for each voxel, and mALFF should have a value of approximately 1 (Zang et al., 2007). In this study, ALFF and fALFF analysis was performed under the slow-5 (0.01–0.027 Hz), slow-4 (0.027–0.073 Hz) and classical frequency band (0.01–0.1 Hz) according to Zuo (Zuo et al., 2010). Then mALFF maps and mfALFF maps of each subject in the three bands were calculated using *REST V1.8*^2^ software based on the MATLAB platform to prepare for the subsequent statistical analysis.

### Classifiers

To assess the diagnostic efficacy of neuronal spontaneous activity, average mALFF values and mfALFF values of 116 brain regions divided based on AAL (Anatomical Automatic Labeling) template under three frequency bands were extracted as the whole brain features. A classifier analysis was performed to investigate the accuracy of ALFF, fALFF and the multimodal fusion of ALFF and fALFF with all features. We measured the separate accuracy of each two groups (d-AD vs. NC, aMCI vs. NC, SCD vs. NC, d-AD vs. aMCI, d-AD vs. SCD, aMCI vs. SCD) with ALFF, fALFF and the multimodal fusion of ALFF and fALFF features, respectively.

A cross-validation was applied to divide the sample data set into two complementary subsets, one for training (classifiers) as a training set, and the other for verifying the validity of the analysis as a testing set. The classifier applied SVM with a linear kernel. The SVM yielded a maximal-margin hyperplane in the feature space, which separated the groups in a training data set. K-fold cross-validation was performed to reduce the variability of the cross-validation results. This cross-validation encompassed the feature selection as well as the classifier. We performed feature selection based on elastic net model (Huang et al., 2018). Elastic net is a linear regression model using L1 and L2 as feature selection parameters. Specially, a multimodal fusion based on SVM was applied to evaluate the classification effect by using a combination of ALFF and fALFF features. In each experiment, we employed inner iterations to determine the feature selection parameters, the model parameters and the modality weights β in the multi-kernel SVM. To further avoid possible biases during partitioning, we repeated the experiments 10 times.

A multimodal SVM adequately utilized the particular characteristics of each modality’s data and provided more possibilities to choose a suitable weighted combination. The kernel function was defined as follows:

(1) $$K(x_{i},\;x_{j})=\langle \Phi (x_{i}),\;\Phi (x_{j})\rangle$$

The kernel function is *K*; *x*_i_, *x*_j_ are the input vectors; and Φ is a map to transform the source data from the input space to feature space. The final kernel function combined with the multimodal data source with a weight coefficient has a form of

(2) $$K(x_{i},\;x_{j})=\beta_{1}K_{1}(x_{i}^{1},\;x_{j}^{1})+\;\beta_{2}K_{2}(x_{i}^{2},\;x_{j}^{2})$$

where {β_n_} is the weight coefficient, and *K*_n_ is the kernel function of each modality’s data *x*^n^. *M* data samples and two modality kernels are used in the learning. The decision function in the classification with a best parameter set is defined as follows:

(3) $$\hat{y}(x)=\sum_{m=1}^{M}\alpha_{m}y_{m}K(x,\;x_{m})+b$$

where {α_m_} is a weight series, *y*_m_ is the label of the sample *x*_m_, *K* is the final kernel defined previously, and *b* is a constant coefficient. It can be noted that the *K* is different from that in equation (2) because it was used for prediction. The SVM algorithm in our study was based on the *LIBSVM* library toolbox^3^ (Chang and Lin, 2011) within the MATLAB environment.

The accuracy (percentage of participants detected correctly), sensitivity (percentage of patients detected correctly), and specificity (percentage of controls detected correctly) for each classifier was calculated to quantify the classification performance. The classification accuracy reflected the predictive power of the algorithm and was of direct diagnostic relevance. In addition, the area under the ROC curve (AUC) was also drawn to evaluate the overall performance of the classification method. The larger the AUC value, the better was the classification performance of the classification method.

### Statistical Analysis

Analysis of covariance (ANCOVA) was used to analyze differences in mALFF/mfALFF throughout the brain based on the voxel level among NC, SCD, aMCI, and d-AD groups. Age, gender, education, mean GM volumes and mean FD were taken as covariates. In the study, we used a GM mask to exclude activities originating from white mater for analyzing ALFF/fALFF differences. All the statistical maps were corrected for multiple comparisons by GRF correction combining the voxel *P*-value < 0.001 and cluster level < 0.05 in DPABI_V3.*0_171210*^4^ based on the Gaussian Random Field Theory.

The clusters showing significant differences were saved as ROIs. We extracted mALFF values from the four groups of subjects for these ROIs using the *REST V1.8* toolkit. *Post hoc* comparisons were then conducted within these ROIs with *SPSS 23.0*, and the two groups with significant differences (*P* < 0.05, *P* < 0.01, *P* < 0.001) were marked. Correction for the *post hoc* comparisons was performed using Bonferroni correction. The fALFF analysis was performed similarly to the ALFF analysis.

### Relationship With Neuropsychological Tests

To test the clinical significance of these ROIs above, we correlated mALFF or mfALFF in these ROIs with neuropsychological tests across all participants including NC, SCD, aMCI, and d-AD. Age, gender, education level, mean GM volumes, mean FD and diagnosis were included as covariates. Bonferroni correction was used to account for multiple comparisons in correlation analyses (*P* < 0.05/5).

## Results

### Demographics and Neuropsychological Test Results

Fifty-seven NC, 44 SCD, 55 aMCI, and 47 d-AD subjects were finally enrolled in this study after excluding subjects with poor registration and restricting head motion to less than 3 mm or 3 degrees. Table 1 summarizes the demographic characteristics and neuropsychological performance of the four groups. No significant group differences were found in gender and mean FD (*P* > 0.05). Age, education, mean GM volume and all cognitive variables showed significant differences between at least two groups (Table 1 and Supplementary Table S1). The d-AD and aMCI performed significantly worse than NC and SCD in all tests. The best memory performance was observed in NC, with intermediate performance in SCD, worse performance in aMCI, and the worst performance in d-AD.

**Table 1:** Demographics and clinical characteristics of the participants.

Demographic data	NC (*n* = 57)	SCD (*n* = 44)	aMCI (*n* = 55)	d-AD (*n* = 47)	*P*-value	*Post hoc*^d^
Gender (male/female)	22/35	19/25	27/28	15/32	0.345^a^	
Age (years)	63.77 ± 8.09	65.13 ± 8.57	67.51 ± 9.62	70.99 ± 10.07	0.001^b^	NC < d-AD, SCD < d-AD
Education (years)	11.05 ± 4.92	11.80 ± 4.65	10.13 ± 4.98	8.89 ± 5.75	0.039^b^	SCD > d-AD
Mean GM volume (L)	0.60 ± 0.07	0.59 ± 0.07	0.56 ± 0.07	0.49 ± 0.05	0.000^b^	NC > aMCI > d-AD,SCD > d-AD
AVLT-I	9.16 ± 1.91	8.27 ± 1.79	6.15 ± 1.71	3.59 ± 1.61	0.000^c^	NC > SCD > aMCI > d-AD
AVLT-D	10.19 ± 2.78	8.50 ± 2.72	4.06 ± 2.88	1.00 ± 1.64	0.000^c^	NC > SCD > aMCI > d-AD
AVLT-R	12.05 ± 2.55	10.96 ± 2.73	7.96 ± 3.74	3.73 ± 3.39	0.000^c^	NC,SCD > aMCI > d-AD
MMSE	28.14 ± 2.13	27.93 ± 1.86	24.66 ± 4.20	16.55 ± 6.21	0.000^c^	NC, SCD > aMCI > d-AD
MoCA	26.10 ± 3.12	25.17 ± 2.91	19.77 ± 4.30	12.55 ± 5.11	0.000^c^	NC > SCD > aMCI > d-AD
Framewise displacement (FD)	0.25 ± 0.12	0.21 ± 0.12	0.25 ± 0.15	0.27 ± 0.13	0.216^b^	


*P < 0.05 means significance existed between the groups. ^*a*^P-value for sex distribution obtained by the chi-square test; ^*b*^P-values obtained by ANOVA. ^*c*^All clinical/cognitive variables from ANCOVA with age, gender, education, mean GM volume, mean FD as covariates; ^*d*^Post hoc testing of cognitive variables based on means adjusted for age, gender, education, mean GM volume and mean FD.*

### Classifiers

**FIGURE 1:**
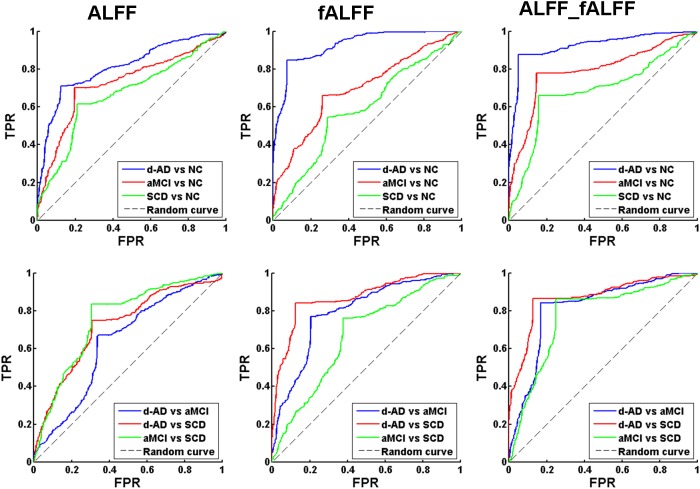
Receiver operating characteristic (ROC) curve of ALFF, fALFF, and ALFF combined with fALFF. TPR, true positive rate; FPR, false positive rate; NC, normal controls; SCD, subjective cognitive decline; aMCI, amnestic mild cognitive impairment; d-AD, dementia of Alzheimer’s disease.

The classifier model performance and ROC curves were depicted in Table 2 and Figure 1. As shown in Table 2, cross-validation of the classifier using ALFF features yielded an accuracy of 80.20% for d-AD vs. NC, followed by an accuracy of 75.36% for aMCI vs. NC and 71.38% for SCD vs. NC. The classifier using fALFF features achieved an accuracy of 89.09, 70.14, and 63.81% for d-AD, aMCI and SCD from NC, respectively. A higher classification effect emerged after fusing ALFF and fALFF features based on multimodal fusion. The separate classifying accuracy of d-AD vs. NC, aMCI vs. NC, and SCD vs. NC was 91.83, 81.94, and 76.44%, respectively.

**Table 2:** Accuracy of the ALFF and fALFF analyses.

Accuracy (AUC)	ALFF	fALFF	ALFF_fALFF
d-AD vs. NC	80.2000(0.8142)	89.0909(0.9276)	91.8273(0.9261)
aMCI vs. NC	75.3561(0.7278)	70.1439(0.6981)	81.9394(0.7933)
SCD vs. NC	71.3818(0.6706)	63.8091(0.5890)	76.4364(0.6871)
d-AD vs. aMCI	66.6727(0.6363)	78.2727(0.7944)	83.8364(0.8182)
d-AD vs. SCD	72.1111(0.7221)	85.9444(0.8720)	87.0778(0.8724)
aMCI vs. SCD	77.4111(0.7561)	70.0333(0.6658)	81.4111(0.7737)


*NC, normal controls; SCD, subjective cognitive decline; aMCI, amnestic mild cognitive impairment; d-AD, dementia of Alzheimer’s disease; AUC, area under the receiver operating characteristic curve.*

The AUC scores were 0.81, 0.73, and 0.67, respectively, for the classification of d-AD vs. NC, aMCI vs. NC, and SCD vs. NC with ALFF features. AUC scores were acquired for the distinction of d-AD, aMCI, and SCD from NC (0.93, 0.70, and 0.59, respectively) by using fALFF features. When combined ALFF and fALFF features via the multimodal fusion, better AUC scores were achieved (AUC = 0.93, 0.79, and 0.69 for d-AD, aMCI and SCD from NC, respectively).

To verify that the identified features are capable of separating patients in different stages, we also constructed the classifier between patient groups (d-AD vs. aMCI, d-AD vs. SCD, aMCI vs. SCD). We found ALFF and fALFF showed different classification results in each of these classifications. ALFF showed higher accuracy in classifications between these groups including aMCI vs. NC, SCD vs. NC, and aMCI vs. SCD, while fALFF showed higher accuracy in other classifications (d-AD vs. NC, d-AD vs. aMCI, and d-AD vs. SCD). However, the multimodal fusion of ALFF and fALFF showed higher accuracy in all classifications than just using ALFF or fALFF separately (Table 2 and Figure 1). Thereby, ALFF and fALFF can’t replace each other, and we investigated both of ALFF and fALFF index among the four groups.

### ALFF/fALFF Analyses Under Different Frequency Bands

**FIGURE 2:**
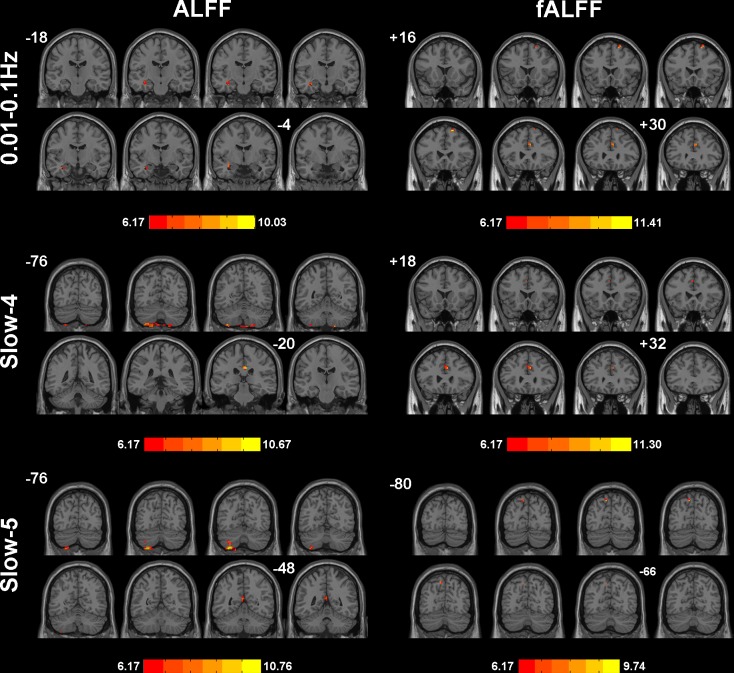
Significant differences of ALFF/fALFF among NC, SCD, aMCI, and d-AD under different frequency bands. The results were obtained by ANCOVA analysis adjusted with mean age, gender, education, mean GM volume and mean FD (*P* < 0.001, cluster level < 0.05, GRF correction) by *DPABI_V3.0_171210*.

We selected clusters with significant differences and labeled their corresponding anatomical location, MNI coordinates, intensity of the significance, Brodmann and AAL partition (Table 3). These clusters were divided into several brain regions according to Andrews-Hanna et al. (2014). The results were shown in Figure 2.

**Table 3:** ANCOVA results with age, gender, education, mean GM volume and mean FD as covariates across the four groups under three frequency bands.

Frequency bands		Anatomical area	BA	AAL	Peak MNI	Cluster Size	Peak intensity
ALFF	Full	**Medial Temporal Lobe**					
		Hippocampus_L (aal)	20	37	-36,-9,-21	16	7.697
		**Lateral frontal cortex**					
		Frontal_Mid_Orb_R (aal)	11	10	24,66,-9	17	8.0164
		**Posterior cingulate cortex/precuneus**					
		Precuneus_R (aal) extend to Posterior Cingulate	23	68	3,-57,24	11	7.6223
		**Cerebellum regions**					
		Cerebelum_8_R (aal)	0	104	18,-51,-60	145	9.5659
	Slow-4	**Posterior cingulate cortex**					
		Cingulum_Mid_R (aal)	23	34	3,-27,30	15	10.6692
		**Cerebellum regions**					
		Cerebelum_8_L (aal)	0	103	-36,-63,-57	140	9.5891
	Slow-5	**Posterior cingulate cortex/precuneus**					
		Precuneus_L (aal) extend to Posterior Cingulate	30	67	-3,-51,15	11	7.8118
		**Cerebellum regions**					
		Cerebelum_8_L (aal)	0	103	-33,-69,-57	67	10.7576
fALFF	Full	**Anterior cingulate cortex**					
		Cingulum_Ant_R (aal)	24	32	6,27,30	8	8.9079
		**Lateral frontal cortex**					
		Frontal_Sup_R (aal)	8	4	18,24,60	12	11.4105
	Slow-4	**Anterior cingulate cortex**					
		Cingulum_Ant_R (aal)	24	32	6,27,30	12	10.2268
		**Medial frontal cortex**					
		Paracentral_Lobule_R extend to Medial Frontal Gyrus	4	70	12,-30,60	9	11.2975
	Slow-5	**Precuneus**					
		Precuneus_L (aal)	7	67	-9,-75,48	10	8.1812


*Full frequency band (0.01–0.1 Hz); slow-4 band (0.027–0.073 Hz); slow-5 band (0.01–0.027 Hz); BA, Brodmann area; AAL, anatomical automatic labeling; MNI, Montreal Institute of Neurology, Standard Head Anatomical Template; Hippocampus_L (aal): left hippocampus; Frontal_Mid_Orb_R (aal): right orbital part of middle frontal gyrus; Precuneus_R (aal): right precuneus; Cerebelum_8_R (aal): a part of right posterior cerebellum; Cingulum_Mid_R (aal): right median cingulate and paracingulate gyri; Cingulum_Ant_R (aal): right anterior cingulate and paracingulate gyri; Frontal_Sup_R (aal): right dorsolateral superior frontal gyrus; Paracentral_Lobule_R: right paracentral lobule; L., left; R., right.*

**FIGURE 3:**
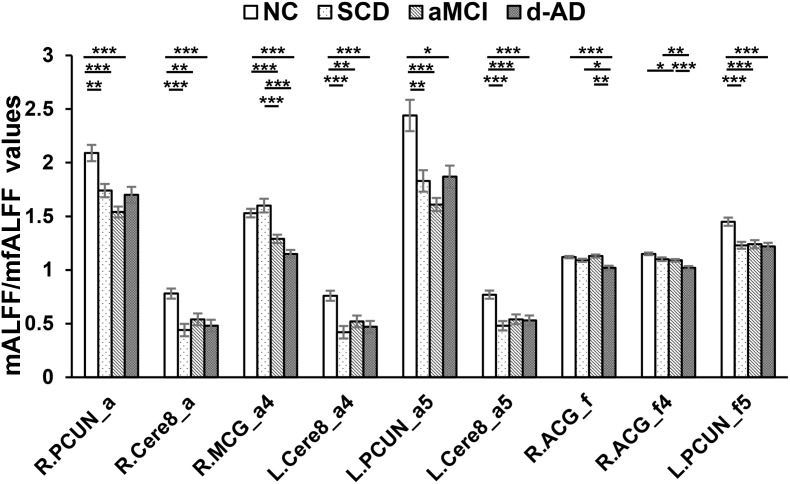
Decreased ALFF and fALFF patterns tendency in patient groups. ^∗^ represents significant level *P* < 0.05, ^∗∗^ means significant level *P* < 0.01, and ^∗∗∗^ means significant level *P* < 0.001. L., left; R., right; R.PCUN, Precuneus_R (aal); R.Cere8, Cerebelum_8_R (aal); R.MCG, Cingulum_Mid_R (aal); L.PCUN, Precuneus_L (aal); R.ACG, Cingulum_Ant_R (aal). ALFF analysis in full band (0.01–0.1 Hz); a4, ALFF analysis in slow-4 band (0.027–0.073 Hz); a5, ALFF analysis in slow-5 band (0.01–0.027 Hz); f, fALFF analysis in full band; f4, fALFF analysis in slow-4 band; f5, fALFF analysis in slow-5 band.

**FIGURE 4:**
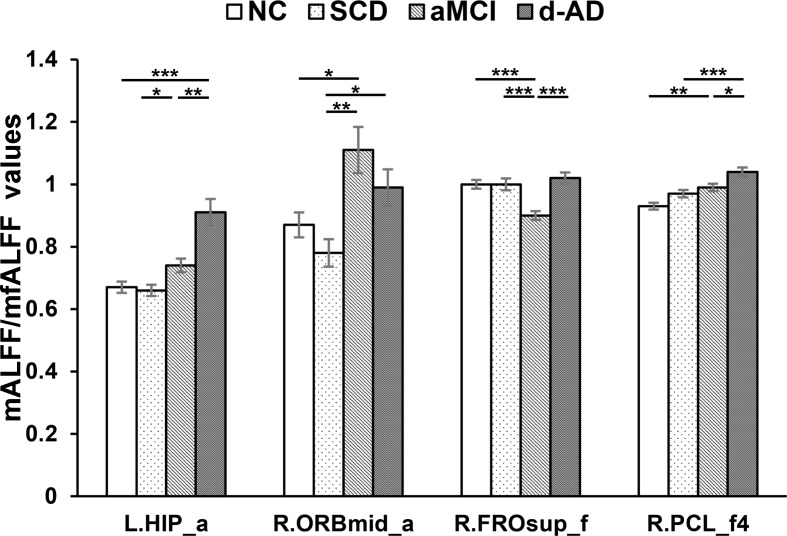
Increased ALFF and fALFF patterns tendency in patient groups. ^∗^ represents significant level *P* < 0.05, ^∗∗^ means significant level *P* < 0.01, and ^∗∗∗^ means significant level *P* < 0.001. L., left; R., right; L.HIP, Hippocampus_L (aal); R.ORBmid, Frontal_Mid_Orb_R (aal); R.FROsup, Frontal_Sup_R (aal); R.PCL, Paracentral_Lobule_R. a, ALFF analysis in full band (0.01–0.1 Hz); a4, ALFF analysis in slow-4 band (0.027–0.073 Hz); a5, ALFF analysis in slow-5 band (0.01–0.027 Hz); f, fALFF analysis in full band; f4, fALFF analysis in slow-4 band; f5, fALFF analysis in slow-5 band.

During the analysis of ALFF, significant group differences were identified including the Hippocampus_L (aal), Frontal_Mid_Orb_R (aal), Precuneus_R (aal) extend to Posterior Cingulate, and Cerebelum_8_R (aal) in the full band (0.01–0.1 Hz). In the slow-4 band (0.027–0.073 Hz), the Cingulum_Mid_R (aal) and Cerebelum_8_L (aal) were identified. In the slow-5 band (0.01–0.027 Hz), significant group differences were observed in the Precuneus_L (aal) extend to Posterior Cingulate and the Cerebelum_8_L (aal) (Figures 3, 4).

In the fALFF analysis, significant group differences were observed primarily in the Cingulum_Ant_R (aal) and Frontal_Sup_R (aal) in the full band. In the slow-4 band, the Cingulum_Ant_R (aal) and Paracentral_Lobule_R extend to Medial Frontal Gyrus showed significant group differences. In the slow-5 band, only the Precuneus_L (aal) manifested significant differences after GRF correction (Figures 3, 4).

Several brain regions presented a decreasing trend of mALFF/mfALFF values in the four groups, including the Cingulum_Mid_R (aal), bilateral inferior Cerebellum lobe, bilateral precuneus and the Cingulum_Ant_R (aal) as disease progressed (Figure 3).

Compared with NC, SCD showed significantly decreased ALFF levels in the Precuneus_R (aal) and Cerebelum_8_R (aal) in the full band; the Cerebelum_8_L (aal) in the slow-4 band; and decreased mALFF values in the Precuneus_L (aal) and Cerebelum_8_L (aal) in the slow-5 band. SCD also presented significantly decreasing mfALFF values in the Precuneus_L (aal) in the slow-5 band compared to NC. Compared to NC, aMCI performed similar to SCD in regions with significantly decreased mALFF values, with additional Cingulum_Mid_R (aal) in the slow-4 band. The aMCI also presented significantly decreasing mfALFF values in the Frontal_Sup_R (aal) in the full band; Cingulum_Ant_R (aal) in the slow-4 band; and the Precuneus_L (aal) in the slow-5 band compared to NC. Compared to NC, d-AD performed similarly to the aMCI in brain regions with significantly decreasing mALFF values. The d-AD also presented significantly decreasing mfALFF values in the Cingulum_Ant_R (aal) in the full band; Cingulum_Ant_R (aal) in the slow-4 band; and the Precuneus_L (aal) in the slow-5 band. Compared with SCD, LFOs decreased significantly in aMCI in several ROIs including the Frontal_Sup_R (aal) and Cingulum_Mid_R (aal); in Cingulum_Mid_R (aal) and Cingulum_Ant_R (aal) in d-AD. Compared with aMCI, d-AD was observed significantly decreased mfALFF values in the Cingulum_Ant_R (aal) (Figures 3, 4).

After *post hoc* comparisons, we found an increasing trend of mALFF/mfALFF values in the Hippocampus_L (aal), Frontal_Mid_Orb_R (aal), Frontal_Sup_R (aal) and Paracentral_Lobule_R (aal) as disease progressed (Figure 4).

Specifically, aMCI showed significantly higher LFOs in the Frontal_Mid_Orb_R (aal) and the Paracentral_Lobule_R (aal) than NC. The Hippocampus_L (aal) and Paracentral_Lobule_R (aal) presented significantly higher LFOs in d-AD than NC. Compared to SCD, aMCI and d-AD showed significantly increased LFOs in the Hippocampus_L (aal), and the Frontal_Mid_Orb_R (aal). The Hippocampus_L (aal), Frontal_Sup_R (aal) and Paracentral_Lobule_R showed significantly higher LFOs in d-AD, compared with aMCI (Figure 4).

### Correlation With Neuropsychological Tests

We correlated mALFF/mfALFF values of ROIs in Table 2 with behavioral scales controlling for age, gender, education, group, mean GM volumes, mean FD and diagnosis as covariates, and various correlations were detected (Supplementary Table S1). Seven brain regions showed significances. Two regions of fALFF features failed to undergo the Bonferroni correlation analysis (Supplementary Table S1). Here, we tried to analyze degenerative changes in AD by combining existing 5 features (Bonferroni, *P* < 0.05/5) with severity degree and alterations of LFOs.

The mALFF values of the Hippocampus_L (aal) and Cerebelum_8_R (aal) in the full band were significantly negative correlated with recognition of AVLT (AVLT-R) scores (Bonferroni, *P* < 0.05/5). And mALFF values of the Cingulum_Mid_R (aal) in the slow-4 band were significantly positive correlated with delayed recall of AVLT (AVLT-D) and MMSE scores (Bonferroni, *P* < 0.05/5). The mALFF values of the Cerebelum_8_L (aal) were significantly negative correlated with AVLT-R scores in slow-4 band; with AVLT-D and AVLT-R scores in slow-5 band (Bonferroni, *P* < 0.05/5).

**FIGURE 5:**
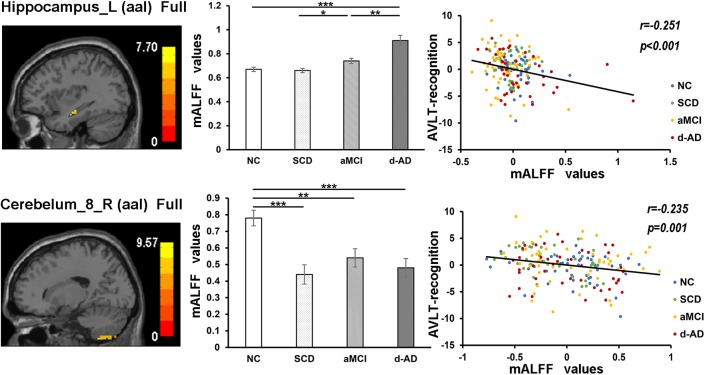
The correlation between mALFF values of the Hippocampus_L (aal) and Cerebelum_8_R (aal) in full band and recognition of AVLT scores. The column 1 showed corresponding anatomic location of the left hippocampus and Cerebelum_8_R (aal) in the brain. The column 2 displayed significant differences of mALFF values of these two regions in full band among four groups obtained by *post hoc* test (Bonferroni correction, *P* < 0.05). ^∗^ represents significant level *P* < 0.05; ^∗∗^ means significant level *P* < 0.01; ^∗∗∗^ means significant level *P* < 0.001. The column 3 were scatter plots demonstrating the negative correlation between the Hippocampus_L (aal) and Cerebelum_8_R (aal) and recognition of AVLT scores (Bonferroni correction, *P* < 0.05/5). Corresponding correlation coefficient “*r*” and significant level “*p*” were marked. NC, normal controls; SCD, subjective cognitive decline; aMCI, amnestic mild cognitive impairment; d-AD, dementia of Alzheimer’s disease; mALFF, the normalized amplitude of the low-frequency fluctuation; AVLT, the auditory verbal learning test.

In the Hippocampus_L (aal) in full band, aMCI showed significantly higher mALFF values than SCD, and d-AD showed significantly higher ALFF levels than NC, SCD, and aMCI. In the Cerebelum_8_R (aal) in full band, SCD, aMCI and d-AD all showed significantly decreased mALFF values than NC (Figure 5).

**FIGURE 6:**
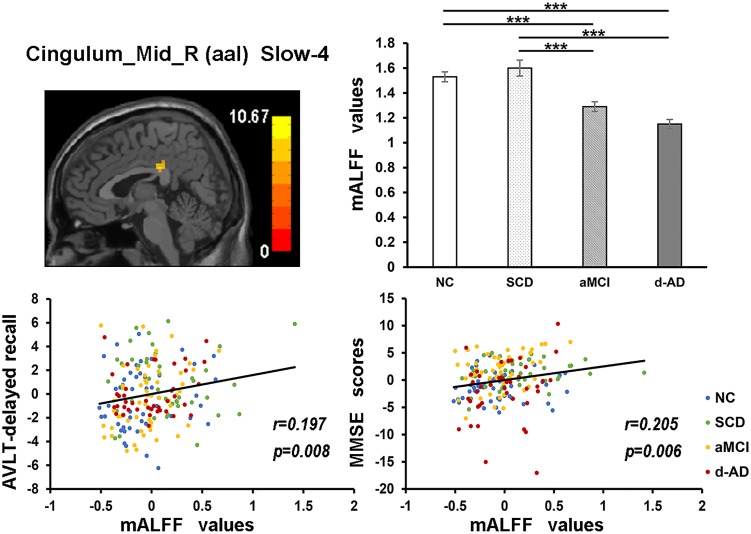
The correlation between mALFF values of the Cingulum_Mid_R (aal) in slow-4 band and delayed recall of AVLT/MMSE scores. The row 1, column 1 showed the corresponding anatomic location of the right median cingulate and paracingulate gyri in the brain. The row 1, column 2 displayed significant differences of mALFF values of this region in slow-4 band among four groups obtained by *post hoc* test (Bonferroni correction, *P* < 0.05). ^∗∗∗^ means significant level *P* < 0.001. The row 2 were scatter plots demonstrating the positive correlation between the right median cingulate and paracingulate gyri and delayed recall of AVLT/MMSE scores (Bonferroni correction, *P* < 0.05/5). Corresponding correlation coefficient “*r*” and significant level “*p*” were marked. NC, normal controls; SCD, subjective cognitive decline; aMCI, amnestic mild cognitive impairment; d-AD, dementia of Alzheimer’s disease; AVLT, the auditory verbal learning test; MMSE, the Mini–Mental State Examination.

In the Cingulum_Mid_R (aal) in slow-4 band, both NC and SCD showed significantly higher mALFF values compared with aMCI and d-AD (Figure 6).

**FIGURE 7:**
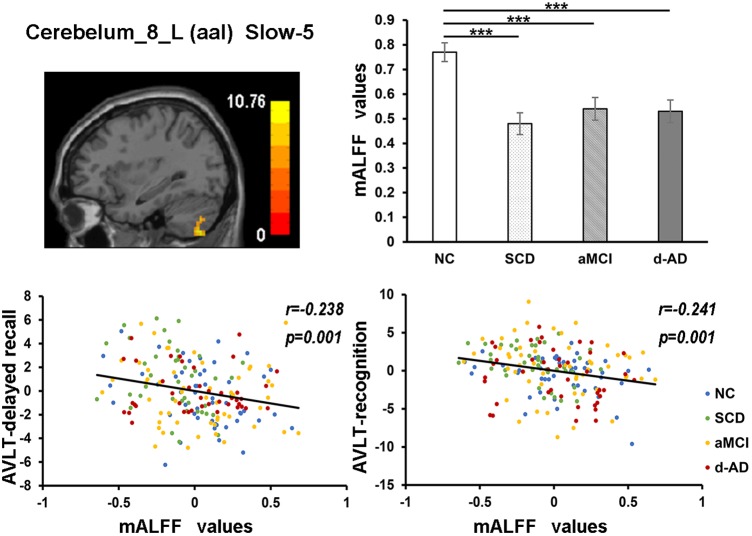
The correlation between mALFF values of the Cerebelum_8_L (aal) in slow-5 band and delayed recall/recognition of AVLT scores. The row 1, column 1 showed the corresponding anatomic location of Cerebelum_8_L (aal) in the brain. The row 1, column 2 described corresponding group comparison obtained by *post hoc* test (Bonferroni correction, *P* < 0.05). ^∗∗∗^ means significant level *P* < 0.001. The row 2 showed negative correlation between this region and delayed recall/recognition of AVLT scores (Bonferroni correction, *P* < 0.05/5). Corresponding correlation coefficient “*r*” and significant level “*p*” were marked. NC, normal controls; SCD, subjective cognitive decline; aMCI, amnestic mild cognitive impairment; d-AD, dementia of Alzheimer’s disease; AVLT, the auditory verbal learning test.

In the Cerebelum_8_L (aal) in slow-4 and slow-5 bands, patient groups all presented significantly lower mALFF values than NC (Figure 7).

## Discussion

In this study, the classifier model based on the fusion of ALFF and fALFF features performed better in the distinction of patients and NC, providing a higher classification accuracy and larger AUC than only using the ALFF or fALFF indexes. We calculated mALFF/mfALFF values in NC, SCD, aMCI, and d-AD at three frequency bands, examined alterations in patient groups compared with NC, and further conducted behavioral correlation analysis with neuropsychological tests. There were widespread differences of mALFF/mfALFF values among these bands in several brain regions in patient groups compared with NC. Some regions with changes of mALFF/mfALFF values presented a similar pattern in patient groups as an increasing or decreasing tendency. Furthermore, there seems to be a gradual pattern in AD spectrum: as the disease progressed, the number of altered brain regions with significantly increased/decreased mALFF/mfALFF values was increasing, and the extent of disruption was enhanced. Several regions with altered mALFF/mfALFF values were significantly correlated with the neuropsychological tests.

### Classifier

The high identification accuracies demonstrated that both ALFF and fALFF could depict spontaneous functional alterations of brain regions in AD spectrum. This procedure was efficient and robust, enabling us to distinguish patients at various stages of AD with high accuracy and AUC values, and indicating that ALFF/fALFF could be a potential index to monitor disease progression. The classification framework composed of the integration of ALFF and fALFF achieved the best performance than only choosing any one of them or simply combined them in the AD staging. The ALFF directly reflect the intensity of regional spontaneous neural activity and potentially more sensitive for discerning differences between groups (Zang et al., 2007). The fALFF was developed after the original ALFF index to detect intrinsic spontaneous brain activity with higher sensitivity and specificity. It can provide a more specific measure of low oscillatory phenomena (Zou et al., 2008, 2010). Overall sensitivity to discriminate brain alterations was stronger for fALFF than ALFF; but the reliability to GM signals was lower for fALFF vs. ALFF (Meda et al., 2015). These two different parameters showed similarities in the main results and differences in the certain brain regions, which may demonstrate an inherent differences in specificity and sensitivity between these two indexes (Zhou et al., 2017). So they were usually applied to the same sample group simultaneously to maximize reliability across subjects and provide sufficient specificity to capture inter-individual differences (Zuo et al., 2010; Zhou et al., 2017). They captured both unique and shared effects across four groups.

### Decreased Tendency of mALFF/mfALFF Values in Patient Groups

As disease progressed, mALFF/mfALFF values presented with decreasing tendency in several brain regions, including the right median cingulate and paracingulate gyri, bilateral inferior cerebellum lobe (belongs to posterior cerebellum), bilateral precuneus, and the right anterior cingulate and paracingulate gyri (Figure 3). These changes may directly imply weakening of the activities of neurons in these regions, potentially due to neurophysiological processes and indicating the presence of a functional deficiency or downregulation of excitability.

These regions all belong to the DMN, which is involved in episodic memory processing, significantly correlated with hippocampal formation and has been consistently associated with the successful recollection of previously studied items (Buckner et al., 2008; Raichle, 2015). Progressive deficits in the DMN were observed in aMCI during a longitudinal follow-up (Bai et al., 2011). Converging evidence indicates that the functional connectivity within the DMN, especially the posterior part, is disrupted in relation to memory impairment in MCI and d-AD patients (Jacobs et al., 2013). Since the precuneus is the putative pivotal region of the DMN and functions as a cortical hub that is highly metabolically active and highly interconnected in the network architecture, it has a particular susceptibility to Alzheimer’s-type neurodegeneration (Drzezga et al., 2011; Tomasi and Volkow, 2011). Previous studies have also detected a specific regional decrease in LFOs of the precuneus in MCI, early d-AD, and d-AD (Zhao et al., 2012; Liu et al., 2014; Huang et al., 2015), and a decline in metabolism in the precuneus at the pre-clinical stage of AD in PET studies (Minoshima et al., 1997). The precuneus was significantly thinner in amyloid-positive MCI than healthy amyloid-negative controls (Rane et al., 2018). SCD has presented significantly decreased mALFF values in the bilateral precuneus and descending mfALFF values in the left precuneus compared with NC, which may represent the existence of abnormity in SCD. Lower path length values were detected in precuneus in SCD, which was associated with a steeper decline in global cognition (Verfaillie et al., 2018). SCD also displayed lower functional connectivity of the precuneus compared with controls without memory complaints (Viviano et al., 2018).

### Increased Tendency of mALFF/mfALFF Values in Patient Groups

In addition, mALFF/mfALFF values presented with incremental tendency were also observed in patient groups in the following brain regions, including the left hippocampus, right orbital part of middle frontal gyrus, right dorsolateral superior frontal gyrus and right paracentral lobule (Figure 4). It may be inferred that with the development of disease, neural damage strengthened the activity in an inverse manner.

The paradoxical increase in mALFF/mfALFF levels may be the result of the amyloid-induced hyper-excitability of neurons and impending neuronal network breakdown as a result of the increasing local and global neurodegenerative pathology (Ewers et al., 2011). The higher mALFF values may be caused by the greater neural activity involved in transmitting information to other regions and lead to greater connectivity among these regions (Di et al., 2013). This phenomenon may represent the most likely compensatory and neuroplasticity mechanism in response to the accumulation of amyloid plaques, in which a greater number of resources is deployed to maintain the normal performance as much as possible as a reaction to the memory loss during the late course of AD (Huijbers et al., 2015). As disease progresses, cognitive processes gradually rely on an increased number of residual healthy synapses and neurons, as well as alternative brain networks (Stern, 2002). Brain areas with increasing activity are recruited as network resources to maintain cognitive functions following the reduced activity in some areas during the course of AD, which may reflect an increase in episodic memory consolidation or retrieval. The enhancement or inhibition of neuronal activities in these brain regions helps to maintain the physiological homeostasis of the whole brain. However, with further disease progression toward d-AD and an augmented pathology, these brain regions become more disturbed, with greater effects on the associated cognitive functions. Thus, patients with increased activation performed worse, reflecting the maladaptation and impairment. This hyperactivity may also be a harbinger of the impending loss of hippocampal function and subsequent rapid clinical decline, which may be present in the early stage of AD (O’Brien et al., 2010; Sperling et al., 2010).

### Correlations Between Neuropsychological Test Scores and mALFF/mfALFF Values

In the correlation analysis, mALFF values of the left hippocampus in full band were significantly negative correlated with AVLT-R scores (Figure 5). The higher mALFF values were related to poor performance in aMCI and d-AD groups, which may also indicate their impairment and maladaptation in different ways (Shinoura et al., 2011). The neuronal spontaneous activity in this region was abnormally strengthened to maintain cognitive performance along with the disease progression. However, it was unable to maintain this function because the compensatory mechanism collapsed, suggesting that the increased spontaneous activity in this region might represent a decompensation related to the increased negative cognitive bias in aMCI and d-AD patients (Lau et al., 2016). The values were lower in SCD compared with NC (without significance), which may be a compensation strategy.

The mALFF values of the right posterior cerebellum lobe in the full band, as well as the left posterior cerebellum lobe in the slow-4 and slow-5 bands were significantly negative correlated with the AVLT-R scores (Figures 5, 7). The mALFF values of the bilateral posterior cerebellum lobe was lower in three patient groups compared to NC and exhibited descending tendency as disease burden got heavy, which indicated compensation in SCD (normal performance) and gradual decompensation in aMCI and d-AD (damaged performance). The cerebellum is involved in cognitive associative learning (Timmann et al., 2010; Buckner, 2013; Keren-Happuch et al., 2014). The posterior cerebellum contributes to complex cognitive operations (Schoch et al., 2006; Tavano et al., 2007; Stoodley and Schmahmann, 2009). The cerebellar alterations were hypothesized to correlate with different forms of cognitive impairment including MCI and d-AD (Thomann et al., 2008). Atrophy of the posterior cerebellum was related to impaired cognitive performance in AD, demonstrating degenerative changes of the cerebellum in AD (Castellazzi et al., 2014). Moreover, alterations of functional connectivity were also reported in MCI and d-AD (Castellazzi et al., 2014).

The mALFF values of the right median cingulate and paracingulate gyri in the slow-4 band were significantly positive correlated with test scores (Figure 6). As disease progressed, the mALFF values tended to decrease. Lower the intrinsic brain activities, lower were the test scores in aMCI and d-AD. This phenomenon implied the disruptions in aMCI and d-AD. Whereas, the ALFF value of this region was incremental in SCD compared with NC (without significance), which may be a compensation mechanism to maintain the normal performance (Sun et al., 2016). The neural compensation were initiated to maintain the impaired neural reserve and then alternate neural networks were recruited to further improve cognitive function (Steffener and Stern, 2012). The cingulum bundle is the main median associative fasciculus, consisting of long associative fibers that connect cortical brain areas and short associative fibers that band cingulate areas. It is one of the principal WM structures transferring anterior–posterior information, which affects its microstructure in AD (Catheline et al., 2010). A strong and specific correlation has been reported between atrophy of the hippocampal formation and the cingulum bundle, indicating that disruption of the cingulum bundle is related to perturbation of the hippocampal formation (Villain et al., 2008).

Three patient groups all exhibited different extents of altered spontaneous activity. And these graded disruptions of the intrinsic brain activity reflected by mALFF/mfALFF levels were detected in SCD, aMCI, and d-AD. These alterations indicate a gradual aggravating physiological pattern of alterations during a limited period of AD-related pathology along the normal aging-SCD-MCI-AD continuum. Mildly altered spontaneous activities presented in SCD might explain their self-perceived cognitive decline and associated subjective complaints prior to noticeable cognitive deficits. From the perspective of correlation analysis, we intended to speculate about the dynamic alterations of spontaneous activity occur in brain regions throughout the course of AD.

Our results indicated that AD should be considered as a disease in which large-scale distributed neural networks are disturbed. This disruption does not focus on changes in a single brain region but on large-scale network alterations or changes in components of these networks (Jacobs et al., 2013). The brain attempts to maintain normal performance via spontaneous regulatory mechanisms, such as the recruitment of additional neurons, as reflected by increasing ALFF/fALFF levels, or a reduction of activity to acclimatize the metabolic demand of other regions in the early stage before pathological invasion get heavy. As the disease burden increases, some regions are invaded by the pathology and fail to perceive the normal intensity of fluctuations, and the increasing/decreasing activation of additional regions can no longer induce a sufficient intensity of brain activity. With advancing disease, greater disturbances in the brain lead to a greater imbalance between activation and performance. The specific change in the pattern of intrinsic brain activity reflected by ALFF/fALFF alterations provides insights into the biological mechanisms of AD. Our results were consistent with the scenario in which progressive changes arise as disease propagated and supplied brain areas that are potentially involved in such degenerative processes (Rasero et al., 2017). Liang et al has found a general linear pattern of d-AD < late MCI < early MCI < NC or d-AD > late MCI > early MCI > NC in several brain regions with increased/decreased ALFF (Liang et al., 2014). Other studies have also detected progressive alterations in the AD continuum, as reflected by GM volume and cortical thickness decline indicators, atrophy degree and rate differences following the order of d-AD > MCI > NC (Ma et al., 2016). Longitudinal research with follow-up information concerning conversion to d-AD is needed to confirm this hypothesis.

### Limitations

Our study has limitations. First, the samples were obtained using a cross-sectional design. Future longitudinal MRI data will be acquired to validate the disruption patterns with disease progression and further probe the classification accuracy of invert and stable patients. The high identification accuracy of patients groups does not mean the high conversion rate to d-AD. Second, we only analyzed rs-fMRI data. In advanced studies, the combination of multimodal neuroimaging and biological information could yield a comprehensive understanding of the progression patterns in AD. Finally, more extensive neuropsychological tests will be utilized to examine more cognitive aspects of patients to further explore the underlying mechanism in the brain.

## Conclusion

In the present study, we observed comprehensive ALFF and fALFF alterations along with a deterioration of memory function in the AD spectrum. Our results indicated that ALFF/fALFF measurements of spontaneous or intrinsic brain activity may be useful to characterize the early and gradient of physiological alterations in AD. The nature and extent of large-scale brain region alterations varies and is aggravated with disease progression in AD. Our findings may help to better understand the relationship between the deterioration in brain spontaneous functional activity and the clinical characteristics of patients in the AD continuum.

## Ethics Statement

Each participant was provided with a written informed consent and signed it prior to any procedures. The research was authorized by the Medical Research Ethics Committee and Institutional Review Board of Xuanwu Hospital, Beijing, China. All the methods were carried out in accordance with the approved guidelines.

## Author Contributions

LY, YY, TY, and YH drafted the manuscript, study concept or design, and Statistical analysis. LY, YY, YW, XH, JL, PC, TY, and YH revised the manuscript for content and analysis or interpretation of data. LY and YH acquisition of data. YH study supervision or coordination. All authors read and approved the final manuscript.

## Conflict of Interest Statement

The authors declare that the research was conducted in the absence of any commercial or financial relationships that could be construed as a potential conflict of interest.

## Footnotes

1 https://www.nitrc.org/frs/downloadlink.php/10441/?i_agree=1&release_id=3694

2 http://www.restfmri.net/forum/REST_V1.8

3 http://www.csie.ntu.edu.tw/~cjlin/libsvm

4 http://rfmri.org/dpabi

## Abbreviations

AD: Alzheimer’s disease
ADL: Activity of Daily Living
ALFF: amplitude of the low-frequency fluctuation
aMCI: amnestic MCI
ANCOVA: analysis of covariance
AUC: the area under the receiver operating characteristic curve
AVLT: auditory verbal learning test
AVLT-D: AVLT-delayed recall
AVLT-I: AVLT-immediate recall
AVLT-R: AVLT-recognition recall
BOLD: blood oxygenation level-dependent
CDR: Clinical Dementia Rating Scale
d-AD: AD dementia
DMN: default-mode networks
fALFF: fractional ALFF
GM: gray matter
HAMD: Hamilton depression rating scale
HIS: Hachinski Ischemic Scale
LFOs: low-frequency oscillations
mALFF: normalized ALFF
MCI: mild cognitive impairment
MMSE: Mini–Mental State Examination
MoCA: Montreal Cognitive Assessment
MPFC: medial prefrontal cortex
NC: normal controls
PCC: posterior cingulate cortex
ROC: receiver operating characteristic
ROIs: regions of interest
rs-fMRI: resting-state functional magnetic resonance imaging
SCD: subjective cognitive decline
SVM: support vector machines
WM: white matter
