# Supplementary material for: Gradual Disturbances of the Amplitude of Low-Frequency Fluctuations (ALFF) and Fractional ALFF in Alzheimer Spectrum
Source: Front Neurosci. 2018 Dec 20;12:975. doi: 10.3389/fnins.2018.00975 (PMC6306691; doi:10.3389/fnins.2018.00975)
Supplement: Supplementary file 1 [file Table_1.DOCX]

**Table 1．**Correlation relationship between low frequency oscillations and neuropsychological scales in ROIs.

| Brain regions | AVLT-I | | AVLT-D | | AVLT-R | | MMSE | | MoCA | |
| --- | --- | --- | --- | --- | --- | --- | --- | --- | --- | --- |
|  | r | *P* | r | *P* | r | *P* | r | *P* | r | *P* |
| L.HIP_a | -0.130 | *0.081* | -0.118 | *0.114* | -0.251 | *0.001^a^* | -0.167 | *0.025^b^* | -0.094 | *0.207* |
| R.ORBmid_a | 0.010 | *0.896* | 0.025 | *0.741* | -0.103 | *0.170* | -0.046 | *0.541* | -0.029 | *0.695* |
| R.PCUN_a | -0.051 | *0.494* | -0.069 | *0.358* | 0.078 | *0.298* | 0.002 | *0.974* | 0.021 | *0.777* |
| R.Cere8_a | -0.097 | *0.194* | -0.168 | *0.024^b^* | -0.235 | *0.001^a^* | -0.162 | *0.030^b^* | -0.114 | *0.127* |
| R.MCG_a4 | 0.132 | *0.077* | 0.197 | *0.008^a^* | 0.164 | *0.028^b^* | 0.205 | *0.006^a^* | 0.166 | *0.026^b^* |
| L.Cere8_a4 | -0.100 | *0.182* | -0.165 | *0.027^b^* | -0.233 | *0.002^a^* | -0.166 | *0.026^b^* | -0.121 | *0.106* |
| L.PCUN_a5 | -0.028 | *0.711* | -0.051 | *0.495* | 0.01 | *0.892* | -0.06 | *0.425* | -0.066 | *0.381* |
| L.Cere8_a5 | -0.114 | *0.129* | -0.238 | *0.001^a^* | -0.241 | *0.001^a^* | -0.154 | *0.039^b^* | -0.129 | *0.085* |
| R.ACG_f | -0.022 | *0.773* | -0.011 | *0.885* | 0.124 | *0.098* | 0.077 | *0.304* | 0.104 | *0.165* |
| R.FROsup_f | -0.113 | *0.131* | -0.118 | *0.114* | -0.084 | *0.260* | -0.132 | *0.078* | -0.108 | *0.149* |
| R.ACG_f4 | 0.006 | *0.938* | -0.009 | *0.902* | 0.046 | *0.543* | -0.003 | *0.965* | 0.004 | *0.954* |
| R.PCL_f4 | 0.079 | *0.292* | 0.147 | *0.048^b^* | 0.055 | *0.465* | -0.05 | *0.508* | -0.043 | *0.567* |
| L.PCUN_f5 | -0.101 | *0.176* | -0.169 | *0.023^b^* | -0.029 | *0.695* | 0.012 | *0.869* | -0.01 | *0.894* |

Note: AVLT: the auditory verbal learning test; AVLT-I, immediate recall of AVLT; AVLT-R, recognition of AVLT; AVLT-D: delayed recall of AVLT; MMSE: the Mini–Mental State Examination; MoCA: the Beijing version of Montreal Cognitive Assessment; Pearson’s correlation coefficient was reported. Significant level (*P*<0.05) was marked with red color.

^a^ Significant at Bonferroni corrected *P*<0.01 (*P*<0.05/5)

^b^ *P*<0.05
